# Evidence of the adaptive evolution of immune genes in chicken

**DOI:** 10.1186/1756-0500-2-254

**Published:** 2009-12-15

**Authors:** Tim Downing, Paul Cormican, Cliona O'Farrelly, Daniel G Bradley, Andrew T Lloyd

**Affiliations:** 1 Smurfit Institute of Genetics, Trinity College, University of Dublin, Ireland; 2 Current address: Wellcome Trust Sanger Institute, Wellcome Trust Genome Campus, Hinxton, UK; 3 School of Biochemistry and Immunology, Trinity College, University of Dublin, Ireland; 4 Current address: Institute of Molecular Medicine, Trinity College, University of Dublin, Ireland

## Abstract

The basis for understanding the characteristics of gene functional categories in chicken has been enhanced by the ongoing sequencing of the zebra finch genome, the second bird species to be extensively sequenced. This sequence provides an avian context for examining how variation in chicken has evolved since its divergence from its common ancestor with zebra finch as well as well as a calibrating point for studying intraspecific diversity within chicken. Immune genes have been subject to many selective processes during their evolutionary history: this gene class was investigated here in a set of orthologous chicken and zebra finch genes with functions assigned from the human ortholog. Tests demonstrated that nonsynonymous sites at immune genes were highly conserved both in chicken and on the avian lineage. McDonald-Kreitman tests provided evidence of adaptive evolution and a higher rate of selection on fixation of nonsynonymous substitutions at immune genes compared to that at non-immune genes. Further analyses showed that GC content was much higher in chicken than in zebra finch genes, and was significantly elevated in both species' immune genes. Pathogen challenges are likely to have driven the selective forces that have shaped variation at chicken immune genes, and continue to restrict diversity in this functional class.

## Background

Understanding the evolutionary patterns of variability in gene classes can illuminate their functional characteristics. In particular, immune system genes are subject to acute selective pressures in order to resist pathogenic attacks and consequently undergo many protein-level sequence changes. It is known that chicken (*Gallus gallus*) host defence genes evolve under stronger positive selection than other functional categories of genes: in alignments with human genes, they possess lower sequence conservation [1]. In mammals and insects, genes implicated in immunity have higher diversity at non-synonymous relative to synonymous sites [2,3]. In humans, genes associated with defence have a higher fraction of genes subject to positive selection than average, and genes with high rates of nonsynonymous mutations are more frequently associated with disease (Bustamante et al. 2005).

The ongoing sequencing of the zebra finch (*Taeniopygia guttata*) genome provides an avian contrast for the chicken. The lower sequence divergence of the chicken with the zebra finch compared to that with mammalian genomes permits a more precise analysis of functional diversity [4]. Consequently, exploring the evolutionary history of chicken immune genes within the avian lineage is more likely to inform on molecular traits that distinguish them from other genes.

Higher GC content in the chicken genome is associated with smaller chromosome sizes [5] and with higher rates of nucleotide substitution [6]. GC content in the chicken genome is elevated in regions that are gene dense, a trait shared with mammalian genomes [7]. However, the evolution of the chicken genome is less typical because it has been subject to more complex pressures, such as a metabolic incentive to dramatically reduce genome size [8,9]. Therefore avian genomes are likely to be subject to selective processes to optimise their sizes, chromosome structures and gene distributions.

In this study, we analyse a set of zebra finch-chicken gene pairs whose functions were inferred from orthologous human sequences. Intraspecific data on chicken nucleotide variation was combined with the functionally annotated gene pairs so that tests could be conducted for the presence of the selection on immune genes in the avian lineage. Results suggested a more frequent fixation of functional variants in immune genes, in spite of appreciable coding sequence conservation.

## Methods

In order to determine a set of functionally annotated chicken genes, translations of chicken gene transcripts downloaded from the WASHUC1 Ensembl genebuild assembly 2.1 from May 2006 (n = 18,776; http://www.ensembl.org/Gallus_gallus/), most of which were sequenced in [10], were searched against human protein RefSeqs (38,754; http://www.ncbi.nlm.nih.gov/RefSeq) using Blastp [11] to identify single best hit pairs (15,754). These best hits were used as a reference to assign human gene function and process categories from 33,905 Panther human gene entries [12] successfully to 9,910 chicken orthologs.

A published set of 3,653 orthologous chicken-zebra finch protein and coding sequence pairs [13] determined as reciprocal best hits using Blastx [14] and T-Coffee [15] were cross-referenced with the 9,910 chicken genes with orthologous Panther functions to generate 2,604 annotated chicken-zebra finch gene pairs. 64 of these could be identified confidently as those whose human ortholog had a function or process related to immunity. Genes with positions not yet allocated to a defined position on a chromosome were excluded. Only autosomal chromosomes with known chromosome sizes [16] were considered; the Z and W chromosomes have divergent properties and their unique evolutionary history as sex chromosomes may affect the dynamics of immune genes located there [1].

Pairwise ratio *d*_*N*_/*d*_*S *_(*ω*) was calculated for each coding sequence (CDS) alignment using the codeml implementation of the PAML 3.15 package [17] where *d*_*N *_was the number of nonsynonymous mutations per nonsynonymous site and *d*_*S *_the number of synonymous substitutions per synonymous site. If synonymous and nonsynonymous mutations are neutral, the relative rates of each are expected to be equal so that *ω = 1 *[17]. Departures from this, where *ω > 1 *(*d*_*N *_*> d*_*S*_) suggest that nonsynonymous mutations are advantageous, and are maintained under directional selection. If *ω < 1 *(*d*_*N *_*< d*_*S*_) then the nonsynonymous SNPs may be deleterious since they are not preserved and are likely to be subject to purifying selection [17]. GC content at 3^rd ^codon position (GC3) was calculated for each sequence from these alignments.

Intraspecific rates of evolutionary change were also calculated for the 2,604 functionally annotated chicken genes as *P*_*N*_/*P*_*S*_, the ratio of nonsynonymous mutations (*P*_*N*_; which change the amino acid in the protein sequence) to synonymous mutations (*P*_*S*_; which cause no amino acid change) per effective CDS site (calculated as the CDS length corrected for the coverage divided by the gene length). After adjusting for genome sequencing coverage rates, SNP frequencies and GC3 for genes and immune genes were explored using one tailed Student's t-tests and using Pearson's correlation coefficient (r), a measure of the shared linear variation between parameters.

The number of substitutions fixed between chicken and zebra finch at nonsynonymous (*D*_*N*_) and synonymous (*D*_*S*_) sites were determined for the 2,604 genes. McDonald-Kreitman tests [18] were implemented to examine the relative number of differences fixed on the chicken-zebra finch lineage (*D*_*N*_/*D*_*S*_) to the number variable within chicken only (*P*_*N*_/*P*_*S*_). Using Fisher's Exact Test, if *D*_*N*_/*D*_*S *_is significantly greater than *P*_*N*_/*P*_*S*_, it is indicative of non-neutral adaptation in the form of an excess of nonsynonymous changes on the chicken-zebra finch lineage [18]. Because nonsynonymous and synonymous sites are intercalated in the coding sequence, their genealogies are shared and thus the absolute numbers of polymorphisms (*D*_*N*_/*D*_*S*_) can be used instead of the rates (*d*_*N*_/*d*_*S*_) [18].

An observed fixation index (*FI*) for all genes and subsets was also determined as *FI *= *(D*_*N*_/*D*_*S*_)/*(P*_*N*_/*P*_*S*_), reflecting the McDonald Kreitman test. If neutral, *FI *should approximate a value of 1; however, this may be violated in regions of relaxed selective constraint [19]. Consequently, the expected contingency table values of *D*_*N*_, *D*_*S*_, *P*_*N *_and *P*_*S *_for each gene were determined and summed across all genes so that an unbiased and neutral expected fixation index (*eFI*) could be calculated as outlined in [20]. This also allows an estimation of the fraction of nonsynonymous mutations driven by positive selection (*α*) to fixation as *α = (FI - eFI)/eFI*. In addition, the proportion of amino acid-altering substitutions segregating in chicken per gene that were neutral was determined as *f *= *P*_*N*_*L*_*S*_/*P*_*S*_*L*_*N*_, where *L*_*S *_was the total number of synonymous sites and *L*_*N *_was the total number of nonsynonymous sites [19].

## Results

### Conservation at chicken immune genes

The analysis included 410,735 SNPs distributed across the autosomal chicken genome at a rate of 0.011 per kb of transcript covered, a number lower than reported elsewhere [10] because only chicken genes with both zebra finch and functionally annotated human orthologs were investigated. 8,848 of these SNPs were in immune genes: 17 were nonsynonymous and 129 synonymous. In comparison, 1,276 nonsynonymous and 4,940 synonymous SNPs were identified in 401,728 SNPs at non-immune genes. Comparisons of diversity between immune and non-immune genes within chicken showed that the average *P*_*N*_/*P*_*S *_(mean 0.13 for immune vs 0.26 for non-immune; Table 1) was much lower for immune genes, illustrating that nonsynonymous sites within chicken were more conserved at immune genes.

**Table 1:** Mean intra- and inter-specific diversity for chicken and zebra finch at all, immune, non-immune and McDonald-Kreitman tests outlier genes.

Gene set	All	Immune	Non-immune	Genes with p < 0.05^1^
Number	2,604	64	2540	26
*ω*^2^	0.0963 ± 0.130	0.0826 ± 0.091	0.0967 ± 0.131	0.2950 ± 0.169
Chicken GC3	0.600 ± 0.173	0.652 ± 0.171	0.599 ± 0.173	0.518 ± 0.132
Zebra finch GC3	0.554 ± 0.159	0.608 ± 0.182	0.553 ± 0.158	0.507 ± 0.133
*D*_*N*_	94,635	1,504	93,131	1096
*P*_*N*_	1,293	17	1,276	0
*D*_*S*_	384,749	5,852	378,897	1439
*P*_*S*_	5,069	129	4,940	272
*P*_*N *_per kb^3^	0.459	0.327	0.464	0
*P*_*S *_per kb^3^	1.813	2.474	1.800	0.111
*P*_*N*_/*P*_*S*_	0.255	0.132	0.258	0
*D*_*N*_/*D*_*S*_	0.246	0.257	0.246	0.762
*L*_*N*_/*L*_*S*_^4^	3.075	3.363	3.068	2.860
*FI*^5^	0.964	1.950	0.952	0
*eFI*^6^	1.056	0.945	1.060	1.377
*α*^7^	-0.087	1.062	-0.102	-1.000
Coverage^8^	0.814	0.723	0.816	0.859

^1 ^Genes whose McDonald-Kreitman test one tailed p values > 0.05 for *D*_*N*_/*D*_*S *_>*P*_*N*_/*P*_*S*_. ^2 ^Calculation excluded non-immune gene XM_422655 that had *d*_*N *_> 0 and *d*_*S *_= *0*. ^3 ^Per kb of effective CDS nucleotide length. ^4 ^Total number of synonymous (*L*_*S*_) nonsynonymous (*L*_*N*_) sites. ^5 ^Observed fixation index, *FI *= *(D*_*N*_/*D*_*S*_)/*(P*_*N*_/*P*_*S*_). ^6 ^Expected fixation index, *eFI*. ^7 ^Proportion of fixed nonsynonymous mutations driven by positive selection fixed in chicken, *α *= *(FI - eFI)/eFI*.^8 ^Mean transcript coverage per base.

Alignments of chicken and zebra finch genes determined the average *ω *value (0.096), which was about the same as that observed between a red jungle fowl and a broiler for genomic mRNA transcripts (0.098 [21]), and in an analysis of cranially expressed chicken-zebra finch gene pairs (0.085 [20]), indicating that the present dataset was not biased. Mean *ω *values were higher for non-immune (0.097; Table 1) than immune (0.083) genes, signifying conservation of nonsynonymous sites in the avian lineage at immune genes as well.

### Adaptive evolution in the chicken lineage

Genes that had a higher ratio of fixed nonsynonymous to synonymous substitutions (*D*_*N*_/*D*_*S*_) compared to the ratio of segregating nonsynonymous to synonymous substitutions (*P*_*N*_/*P*_*S*_) may be have undergone adaptive evolution [18]. McDonald-Kreitman tests on the set of immune genes showed a significant excess of fixed nonsynonymous changes on the chicken-zebra finch lineage (*FI *= 1.95; one-tailed p = 0.004) that was not present for non-immune genes, whose *FI *value was about two times lower (0.95). *D*_*N*_/*D*_*S *_for non-immune (0.246; Table 1) and immune (0.257) genes were about equal, but *P*_*N*_/*P*_*S *_was much higher for non-immune genes (0.253 vs 0.132 for immune genes). The high number of SNPs per immune gene ensured that this largely unlinked set of loci should be robust to aggregative McDonald-Kreitman tests [2,22]. The mean fraction of neutral amino-acid replacement mutations (*f*) for each gene with *P*_*N*_*> 0 *and *P*_*S*_*> 0 *was not different between those with immune (0.222) and non-immune (0.239) functions.

An unbiased estimate of the neutral rate of the fixation of amino acid changing variants in chicken, *eFI*, was lower for immune (0.95) than non-immune (1.06) genes, further illustrating that immune genes were more conserved than non-immune ones. *eFI *for all genes (1.06) was of the same scale as other datasets [20]. Given the immune set's much higher *FI*, the estimated proportion of amino acid changes fixed in chicken that were driven by positive selection (*α *= *(FI - eFI)/eFI*) was much higher for immune (1.06) than non-immune genes (-0.10). This indicated that immune genes were subject to stronger selective processes and also that there were deleterious alleles present at non-immune genes.

McDonald-Kreitman tests on individual chicken genes identified 26 (1% of the total) with a significantly higher *D*_*N*_/*D*_*S *_than *P*_*N*_/*P*_*S *_(p < 0.05). Although this group had an average *ω *significantly higher than that for all genes (mean 0.295 vs 0.096 for all, p < 1 × 10^-6^; Table 1), no amino acid-altering mutations were found segregating in the chicken population, suggesting that the significant McDonald-Kreitman tests may be detecting strong purifying selection rather than adaptive evolution. This set of genes had an average coverage rate (0.86; Table 1) above that for all genes (0.81), indicating that the absence of the detection of nonsynonymous SNPs segregating in chicken was not due to poorer coverage. This group contained an immunity-related helicase (KU70; McDonald-Kreitman test p = 0.021) and a DNA polymerase (eta; p = 1.8 × 10^-5^) involved in homologous recombination during DNA repair [23] and synthesis [24], respectively.

### GC content higher in immune genes

GC3 was significantly higher for immune genes than for non-immune genes in both chicken (mean 0.65 vs 0.60 for non-immune, p = 0.016) and zebra finch (mean 0.61 vs 0.55 for non-immune, p = 0.006). GC3 was significantly higher for chicken than zebra finch genes (0.60 vs 0.55, p < 1 × 10^-6^; Table 1), though it was highly correlated between the species, as expected (r^2 ^= 0.940, p < 1 × 10^-6^; Additional file 1). Gene rates for GC3 and *ω *did not correlate significantly.

**Figure 1:**
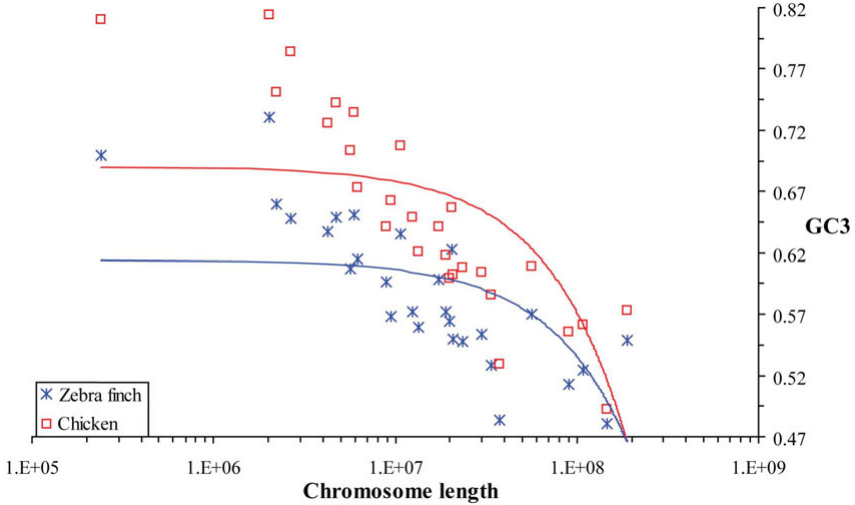
**Correlation of chicken chromosome length with chromosomal GC3 content for chicken genes and their zebra finch orthologs**. The best fitting linear correlations of GC content at the third codon position (GC3) for chicken (red, r^2 ^= 0.435, p = 0.010) and zebra finch (blue, r^2 ^= 0.358, p = 0.030) with chicken chromosome size (on a log scale) are shown by the dashed lines.

Increasing chicken chromosome size correlated with higher chromosomal GC3 rates for chicken genes (r^2 ^= 0.435, p = 0.010; Figure 1) and their zebra finch orthologs (r^2 ^= 0.358, p = 0.030). Although smaller chromosomes tended to have lower chromosomal *ω *values for all genes (r^2 ^= 0.325, p = 0.046; Additional file 2), they had a higher frequency of genic SNPs per kb due to a higher incidence of genes (Additional file 3). This was consistent with previous human-chicken comparison [1] and analyses of SNP diversity [10]. Further F-tests involving chromosomal categories binned in groups according to size suggested that the manner in which these were previously assigned [1] has produced artefactual results; unbinned chromosomes allowed a more robust analysis.

## Conclusions

This study combined an intraspecific analysis of chicken variation and an interspecies survey of chicken and zebra finch genes with orthologous human functions. It demonstrated that amino-acid changing sites immune genes were subject to purifying selection on the avian lineage. The lower rates of polymorphism at immune gene nonsynonymous sites in chicken showed that there was no evidence of a significant relaxation of the selective constraint on chicken immune genes as a group since domestication.

In spite of this, McDonald-Kreitman tests indicated immune genes as a group had a high rate of fixation of nonsynonymous mutations, signifying indicating that they were subject to adaptive evolution on the chicken-zebra finch lineage [18]. This was supported by the high proportion of amino acid changes fixed in chicken for immune genes. A previous study of chicken and zebra finch genes expressed in the brain estimated of the portion of nonsynonymous polymorphisms in chicken that were fixed by positive selection (0.20) [20], indicating that immune genes as a group are under a greater frequency of selective events. The negative *α *value for non-immune genes indicated the incidence of deleterious variants on the chicken-zebra finch lineage [25], which is backed by evidence that a substantial minority (0.23) of amino acid changes segregating in chicken are deleterious [20].

The considerable conservation of nonsynonymous sites at immune genes within chickens has probably exaggerated the perceived strength of positive selection on these sites on the avian lineage [19]. Additionally, it is possible that high recombination or resequencing of rare polymorphisms may inflate this figure [20], and while the chicken's high variability suggests that it has not gone through a major population bottleneck since domestication [10], the fixation of deleterious alleles in tandem with population size increases can amplify estimates of α [26]. Nonetheless, the fraction of fixed replacement substitutions that were under positive selection at chicken immune genes further supports the assertion that this functional category was historically subject to stronger adaptive forces from pathogens and consequently undergoes directional selective sweeps more frequently than other gene groups [2].

McDonald-Kreitman tests suggested that 26 genes were under pervasive purifying selection within chicken. As a group, they had significantly reduced GC content, which is associated with reduced variation [1], and two of these genes were associated with recombination. Lower GC content is associated with decreased recombination [10] implying that the impact of recombination on diversity may necessitate modification of genes controlling this process.

A further examination of GC content showed that it was substantially lower in zebra finch compared to chicken, and significantly higher in immune genes. Chromosome size appeared to be related to *ω *values, suggesting that genes on larger chromosomes may evolve faster, as has been suggested previously [2,27]. Once robust chromosomal assignments of zebra finch genes are established, this could be explored further in order to understand the complex patterns of chromosomal fission, fusion and rearrangements in avian species [28-30] and how this relates to GC content and the evolutionary dynamics of immune genes.

## Competing interests

The authors declare that they have no competing interests.

## Authors' contributions

TD and AL designed the study. TD, PC and AL completed the bioinformatic gene mining and database construction. TD conducted genomic analysis. TD, PC, COF, DB and AL wrote the manuscript.

## Supplementary Material

Additional file 1 **Correlation of GC3 content at chicken and zebra finch genes**. The best fitting linear correlation (not shown) has r^2 ^= 0.94 (p < 1 × 10^-6^). Click here for file

Additional file 2 **Correlation of chicken chromosome size with *ω***. The best fitting linear correlation of chromosome length with chromosomal rates of *ω *= *d*_*N*_/*d*_*S *_is shown by the solid line (r^2 ^= 0.325, p = 0.046). Click here for file

Additional file 3 **Number of SNPs per kb of chicken transcript sequence covered for each chromosome ordered according to decreasing size**. 3' and 5' UTR, indel, frameshift, upstream, downstream, splice site, intronic, exonic and stop-codon SNPs were included. Click here for file
