# Supplementary figures and images for: Evidence of the adaptive evolution of immune genes in chicken
Source: BMC Res Notes. 2009 Dec 15;2:254. doi: 10.1186/1756-0500-2-254 (PMC2804575; doi:10.1186/1756-0500-2-254)

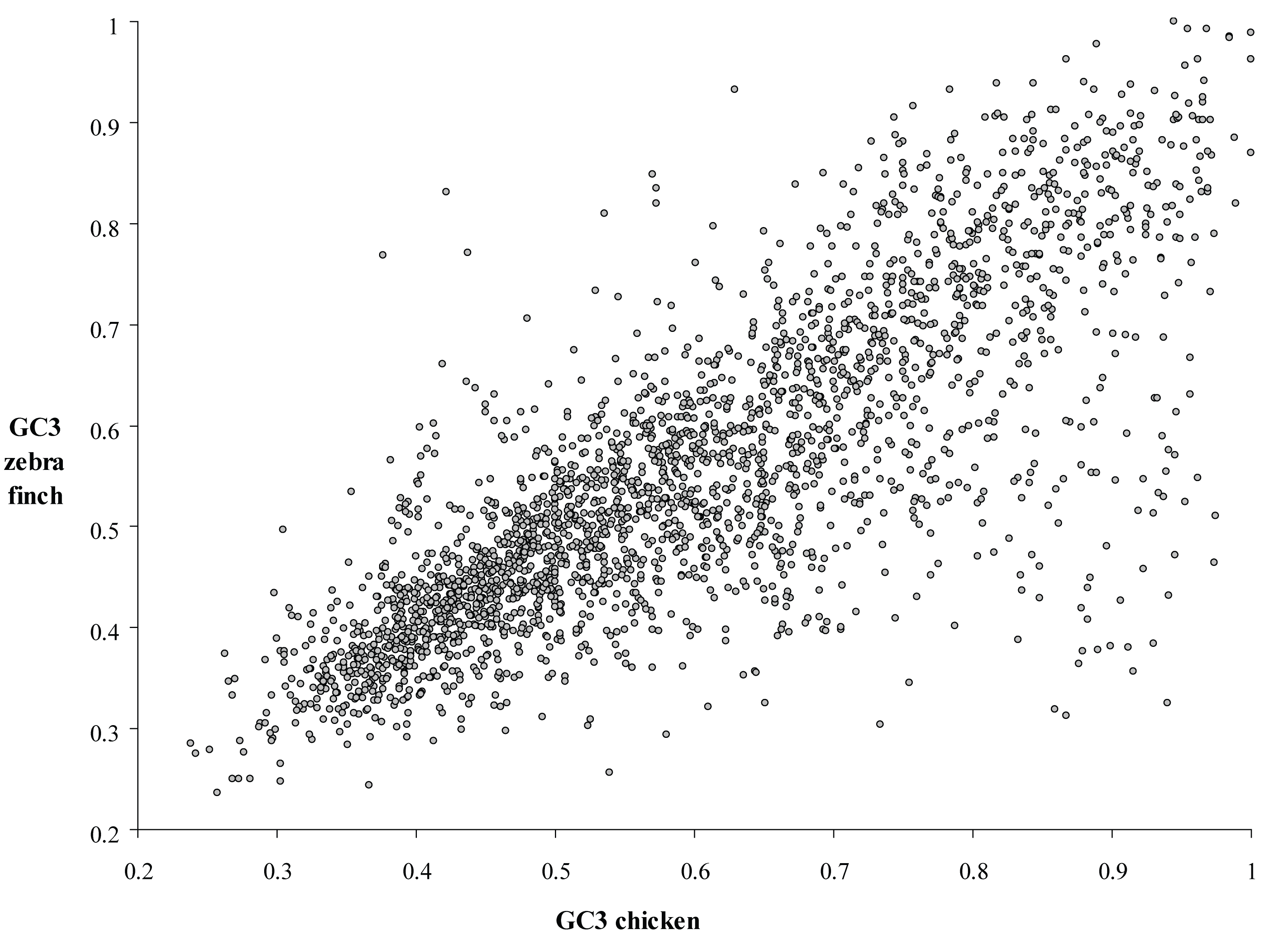

Supplement: Additional file 1 — Correlation of GC3 content at chicken and zebra finch genes. The best fitting linear correlation (not shown) has r2 = 0.94 (p < 1 × 10^-6^). [file 1756-0500-2-254-S1.png]

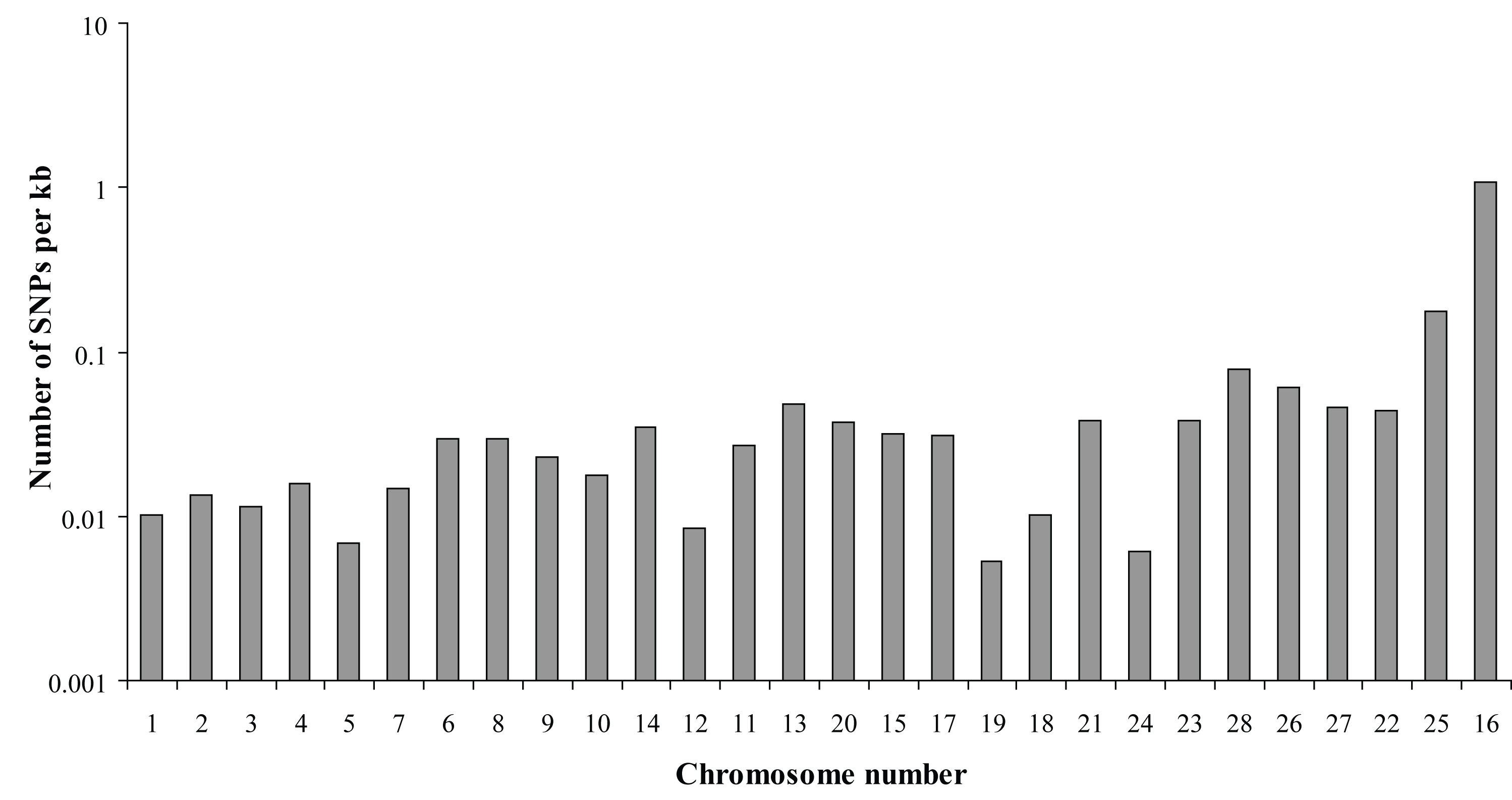

Supplement: Additional file 2 — Correlation of chicken chromosome size with ω. The best fitting linear correlation of chromosome length with chromosomal rates of ω = dN/dS is shown by the solid line (r2 = 0.325, p = 0.046). [file 1756-0500-2-254-S2.png]

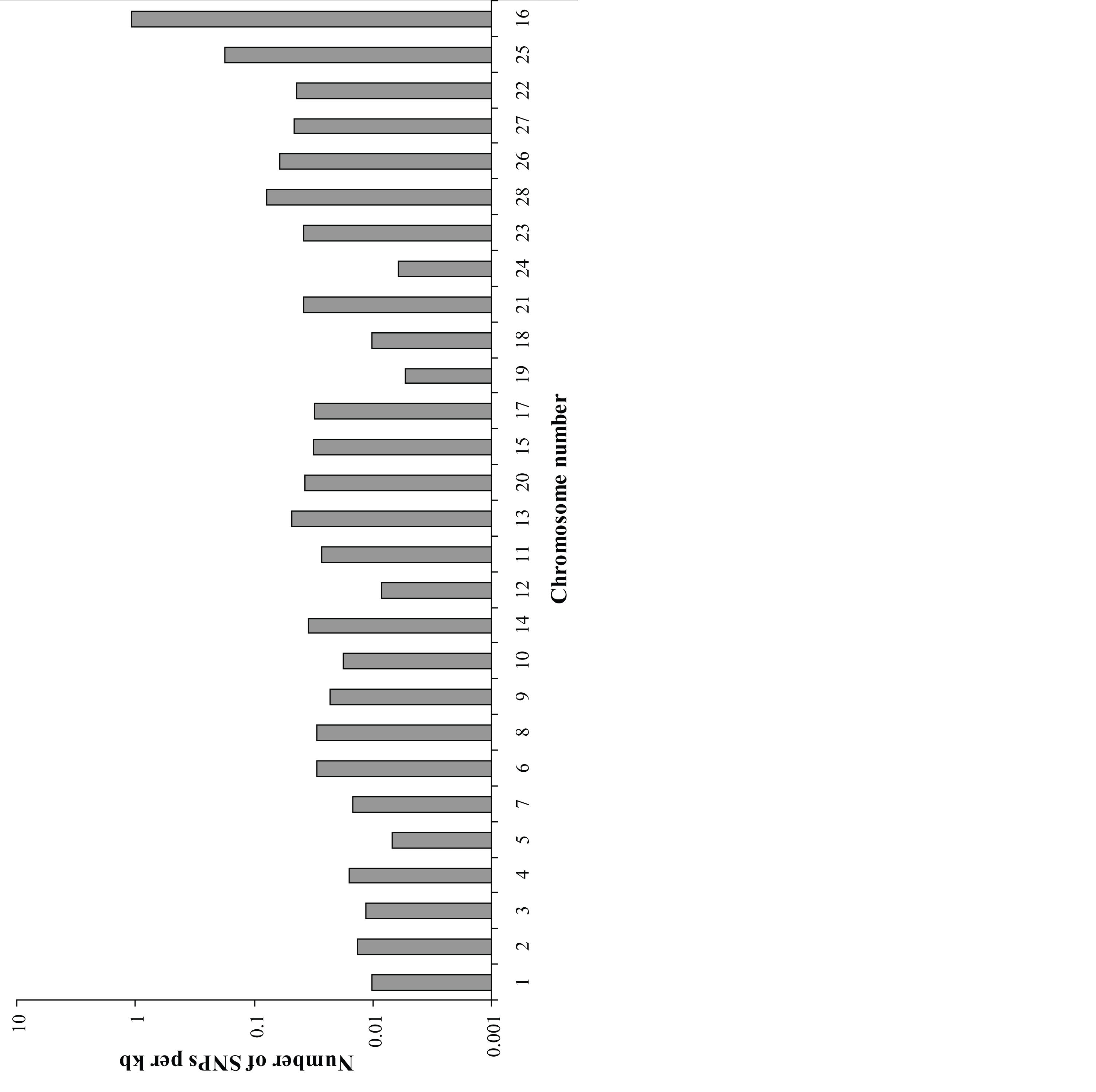

Supplement: Additional file 3 — Number of SNPs per kb of chicken transcript sequence covered for each chromosome ordered according to decreasing size. 3' and 5' UTR, indel, frameshift, upstream, downstream, splice site, intronic, exonic and stop-codon SNPs were included. [file 1756-0500-2-254-S3.png]
